# An R2R3-MYB transcription factor *VyMYB24*, isolated from wild grape *Vitis yanshanesis* J. X. Chen., regulates the plant development and confers the tolerance to drought

**DOI:** 10.3389/fpls.2022.966641

**Published:** 2022-09-08

**Authors:** Ziguo Zhu, Ran Quan, Guangxia Chen, Guanghui Yu, Xiujie Li, Zhen Han, Wenwen Xu, Guirong Li, Jiangli Shi, Bo Li

**Affiliations:** ^1^Shandong Academy of Grape, Shandong Academy of Agricultural Science, Jinan, China; ^2^College of Horticulture and Landscape Architecture, Henan Institute of Science and Technology, Xinxiang, China; ^3^College of Horticulture, Henan Agricultural University, Zhengzhou, China

**Keywords:** MYB genes, *V. yanshanesis*, plant development, drought tolerance, gibberellin

## Abstract

In grapevines, the MYB transcription factors play an important regulatory role in the phenylpropanoid pathway including proanthocyanidin, anthocyanin, and flavonoid biosynthesis. However, the role of MYB in abiotic stresses is not clear. In this study, an R2R3-MYB transcription factor, *VyMYB24*, was isolated from a high drought-tolerant Chinese wild *Vitis* species *V. yanshanesis*. Our findings demonstrated that it was involved in plant development and drought tolerance. *VyMYB24* is a nuclear protein and is significantly induced by drought stress. When over-expressed in tobacco, *VyMYB24* caused plant dwarfing including plant height, leaf area, flower size, and seed weight. The GA1+3 content in transgenic plants was reduced significantly, and spraying exogenous gibberellin could recover the dwarf phenotype of *VyMYB24* transgenic plants, suggesting that *VyMYB24* might inhibit plant development by the regulation of gibberellin (GA) metabolism. Under drought stress, the *VyMYB24* transgenic plants improved their tolerance to drought with a lower wilting rate, lower relative electrical conductivity, and stronger roots. Compared to wild-type tobacco plants, *VyMYB24* transgenic plants accumulated less reactive oxygen, accompanied by increased antioxidant enzyme activity and upregulated gene expression levels of superoxide dismutase (SOD), peroxidase (POD), and catalase (CAT) genes. In addition, transgenic plants accumulated more proline, and their related synthetic genes *NtP5CR* and *NtP5CS* genes were significantly upregulated when exposed to drought. Besides, abiotic stress-responsive genes, *NtDREB, NtERD10C, NtERD10D*, and *NtLEA5*, were upregulated significantly in *VyMYB24* transgenic plants. These results indicate that *VyMYB24* plays a positive regulatory role in response to drought stress and also regulates plant development, which provides new evidence to further explore the molecular mechanism of drought stress of the MYB gene family.

## Introduction

In their lifespan, plants are often subjected to various biotic or abiotic stresses such as drought, salinization, cold, disease, insect pests, etc. Of them, drought stress is the most common. At present, more than one-third of the land area worldwide is located in arid or semi-arid regions (Khresat et al., 2004). Water deficit triggers wilt, restricts plant growth, and inhibits photosynthesis, resulting in significant financial losses. The plant's response to drought is a complex gene expression network. The water deficit signal is transferred to the cell membrane or nucleus, which directly triggers a second messenger system (Ca^2+^/IP3/CDPK phosphorylation and dephosphorylation) and activates the expression of a transcription factor or drought-responsive genes that function in transcriptional regulation and synthesis of protective enzymes and metabolites. This finally causes changes in cell physiological and biochemical processes to adapt to drought stress (Zhu, 2002).

The MYB family is one of the largest transcription factor families in higher plants. There are 190 MYB members in *Arabidopsis thaliana* (Stracke et al., 2001), 157 in maize (Du et al., 2012), 183 in rice (Chen et al., 2006), and 192 in *Populus* (Wilkins et al., 2009). The MYB transcription factor contains a highly conserved DNA binding domain (MYB domain) at the N terminal. According to the number of adjacent MYB domains, MYB transcription factors can be divided into four subfamilies: MYB-related, R2R3-MYB, 3R-MYB, and 4R-MYB (Dubos et al., 2010). Among them, the R2R3-MYB transcription factor has more members and various functions including secondary metabolism (Zhou et al., 2019; Ke et al., 2021), cell differentiation (Lu et al., 2021), and organ morphogenesis (Lau et al., 2015). Increasingly, recent reports show that R2R3-MYB proteins have been found to play an important regulatory role in response to abiotic stresses such as drought, cold, and salinity stresses. In *Arabidopsis, AtMYB60* promoted root elongation to increase water uptake in the early stage of drought, and ABA was involved in drought resistance of *AtMYB60* by regulating stomatal closure (Oh et al., 2011). The *AtMYB15* participated in the response to cold stress by forming the complex *inducer of CBF expression 1* (*ICE1*) and specifically combined it with the MYB element of CBF gene promoter (Agarwal et al., 2006a). In *Oryza sativa, OsMYB30* interacted with the Jasmonate ZIM-domain gene (*OsJAZ9*) to regulate the expression of β-amylase, which degraded starch to maltose and enhanced the tolerance to cold stress as a soluble substance (Lv et al., 2017; Zeng et al., 2019). Abscisic acid (ABA) was involved in the drought tolerance of *OsMYB48-1* by upregulating the expression of the ABA biosynthesis genes *OsNCED4* and *OsNCED5*, as well as the stress-responsive genes *RAB21, OsLEA1, RAB16C*, and *RAB16D* (Xiong et al., 2014). In wheat, the heterologous expression of *TaMyb1D* in tobacco suppressed the expression of the phenylalanine metabolism-related genes and reduced the accumulation of flavonoids and lignin, thus enhancing the tolerance to drought and oxidative stress (Wei et al., 2017).

In grapevines, 108 members of the R2R3 MYB subfamily were identified (Matus et al., 2008). Previous studies on grapevine MYB genes revealed an unexpected wealth of genes. For example, grapevine *MYB14* and *MYB15* genes regulated the stilbene accumulation (Höll et al., 2013; Duan et al., 2016); *VIMYBA1* genes, *VvMYBC2-L1, VvMYB4*, and *VvMYB5a*, modulated anthocyanin and proanthocyanidin biosynthesis (Deluc et al., 2008; Cutanda-Perez et al., 2009; Huang et al., 2014; Perez-Diaz et al., 2016); *VvMYBPA1* and *VvMYB5b* genes induced phenylpropanoid pathway (Deluc et al., 2006; Bogs et al., 2007); and *VvMYBF1* controlled flavonoid synthesis (Czemmel et al., 2009). However, the possible role of grapevine R2R3-MYB in drought stress is not clear.

*Vitis yeshanensis* J. X. Chen. is an important germplasm resource native to North China with high resistance to drought (Cui et al., 2020). In our previous study, a drought stress-induced differential expression gene VIT_14s0066g01090, *VyMYB24*, was screened from the transcriptome of *V. yanshensis*, which was upregulated in response to drought treatment. Based on its specific expression under drought stress, the present study mainly focuses on the potential role of the *VyMYB24* gene in responding to drought tolerance and elaborates on its function in development regulation. Our study aimed to provide valuable information for exploring the more complex functions of grapevine MYB genes and contribute to future grapevine improvement with high drought resistance.

## Materials and methods

### Plant material and stress treatments

*Vitis yeshanensis* accession Yanshan-1 was grown in the grape repository of Shandong Institute of Pomology (Tai'an, Shandong, China). Two-year cutting plants in pots were cultivated in a greenhouse under a 16/8 h photoperiod (2000 lx) and the relative humidity of approximately 70–80% at 25–28°C. The drought treatment was conducted when the soil water content reached 70% in pots, and the leaves were sampled at 0, 1, 2, 3, 4, 5, 7, and 9 days. For salt treatment, the plants were watered with a 0.1 mol/L NaCl solution till flowing from the bottom of the pot. For temperature treatment, the plants were placed in the illumination incubator at 40°C. The leaves were collected at 0, 3, 6, 9, 12, 24, 36, and 48 h after salt treatment and high-temperature treatment, respectively. The leaf samples were frozen with liquid nitrogen and stored at −80°C. Each treatment included six pots of grape plants with a uniform growth trend. The normally managed plants were considered as control (CK).

The tobacco plants (*Nicotiana benthamiana* L.) were sowed *in vitro* on an MS medium under a 16/8 h photoperiod at 25°C. Ten-day-old seedlings were transferred into the soil with pots of 7 cm length, 7 cm width, and 10 cm height. Phenotypic indexes of transgenic plants, such as plant height, leaves, flowers, and fruit, were observed and recorded during different plant development periods. Seed weight was expressed as the mean value of three seeds. For GA treatment, three-week tobacco plants were sprayed using 10^−5^ M GA3 two times at intervals of five days, and the plant height was measured after three weeks.

Four-week-old tobacco plants were chosen to perform the drought treatment without watering under a 16/8 h photoperiod at 28°C. The control plants were watered normally. After 10 days of treatment, the physiological indexes were calculated. On the 12th day after drought stress, the wilting rate was recorded, and the fresh weight of the root was measured. The wilting rate was calculated based on the number of plants with more than 50% leaves with wilting phenotype to the total plants. Each treatment included 90 plants in three biological repeats.

### *VyMYB24* cloning

Total RNA was extracted from Yanshan-1 leaves using E.Z.N.A. total RNA Kit I (OMEGA). The first cDNA was amplified by the SMART MMLV Reverse Transcriptase (TaKaRa). Specific primers were designed according to the DEG VIT_14s0066g01090 MYB gene with the forward primer: 5′-atggataaaaaaccctgcaattctcag-3′ and the reverse primer: 5′-ttaatctccattaagtagctgcatag-3′. The *VyMYB24* gene was cloned using PrimeSTAR^®^ HS DNA Polymerase (Takara, Beijing, China). PCR was performed in 30 cycles of 98°C for 10 s and 60°C for 10 s, followed by 72°C for 10 min. The obtained product was cloned into pGEM-T Easy Vector System (Promega, USA) at 4°C overnight, transformed into *Escherichia coli* strain TOP10 (Tiangen, Beijing, China), screened on the LB plate, and then sequenced by Sangon Biotech Co., Ltd (Shanghai, China).

### Quantitative real-time PCR analysis

Total RNA extraction and cDNA amplification were performed according to the above method. The Quantitative real-time PCR (qRT-PCR) was run on a CFX96 real-time PCR cycler (Bio-Rad Laboratories, Hercules, CA, USA) using SYBR Premix Ex Taq^TM^ II Kit (Takara, Beijing, China), with the following cycling conditions: 30 s at 95°C and 40 cycles of 5 s at 95°C and 30 s at 60°C. The *Actin* gene was used as an internal reference. Relative mRNA ratios were calculated using the 2^−ΔΔCT^ (Livak and Schmittgen, 2001). The specific primers used for qPCR are listed in Supplementary Table 1.

### Subcellular localization of *VyMYB24*

The *VyMYB24* coding region was fused to GFP in the pBI221-GFP plasmid to construct the *VvMYB24*-GFP vector. For subcellular localization assays, *Arabidopsis* protoplasts were isolated according to the previous protocol (Yoo et al., 2007). The vector was transferred to the protoplasts using the PEG6000 method. The transfected protoplasts were incubated for 12–16 h at 28°C in the dark. Fluorescence was detected using a confocal laser scanning microscope (LSM510; Carl Zeiss Thornwood, New York, NY, USA).

### Generation of *VyMYB24* transgenic tobacco plants

The open reading frame sequence of *VyMYB24* was amplified with the forward primer 5′-ggggtaccATGGATAAAAAACCCTGCAATTC-3′ and reverse primer 5′-gggtcgacATCTCCATTAAGTAGCTGCAT-3′. The resulting fragments were digested by restriction endonucleases KpnI and SalI and inserted into the pCAMBIA2300 vector to produce the pCAMBIA2300:: *VyMYB24* construct. These constructs were transformed into *N. benthamiana via Agrobacterium* GV3101 using the leaf disc method according to the protocol described by Horsch et al. (1985). Transgenic tobacco plants were placed on the MS medium with added kanamycin (50 mg·L^−1^). The T2 transgenic tobacco plants were grown in a greenhouse under a 16/8 h photoperiod at 25°C.

### Evaluation of chlorophyll content, relative electrical conductivity, and malondialdehyde (MDA) content

Total chlorophyll concentration was measured according to the method described by Costa et al. (2008). About 0.1 g of leaves were extracted using 80% acetone and analyzed using a UV-Visible Spectrophotometer (UV2600, Shimadzu, Kyoto, Japan). The absorbance was measured at 645 and 663 nm, respectively. For relative electrical conductivity, 0.3 g of fresh leaves were cut into pieces and placed in a tube with 10 ml sterile deionized water for 3 h; the electrical conductivity (R1) was measured with a Crison conductivity meter (Basic 30, Crison Instruments, SA). Then, the samples were bathed in boiling water at 100°C for 20 min and cooled to room temperature. The electrical conductivity (R2) was measured. The relative electrical conductivity was calculated with R1/R2×100%. The MDA content was measured by the thiobarbituric acid (TBA) method described by Zhao et al. (2009) and expressed as U/mg protein.

### Histochemical ROS staining and antioxidant enzyme activity analysis

For 3, 3′-diaminobenzidine (DAB) and tetranitroblue tetrazolium chloride (NBT) staining, the leaves were infiltrated in the DAB solution (0.1%, in 50 nM Tris-HCl, pH 3.8) or the NBT solution (0.5 mg/ml, in 0.25 mM phosphate buffer, pH 7.6). After overnight in dark, the leaf samples were put in 95% alcohol and bathed in boiling water at 100°C for 10 min. After cooling, the leaves were kept in a bleaching solution (lactic acid: glycerin: H_2_O = 1:1:4) and photographed. The H_2_O_2_ and ${\text{O}}_{2}^{-}$ contents were measured by the ammonium molybdate method or hydroxylamine oxidation method described by Kong et al. (2011). For drought tolerance analysis, the enzyme activity of the antioxidant enzyme, including superoxidase dismutase (SOD), peroxidase (POD), and catalase (CAT) in tobacco leaves, were evaluated using A001-1, A084-3, and A007-1-1 kits (Nanjing Jiancheng Co. Ltd, China), respectively.

### Measurement of proline and gibberellin (GA) content

The osmosis substance proline was measured by the ninhydrin method described by Kong et al. (2011), and GA contents were measured by the enzyme-linked immunosorbent assay (ELISA) method as described previously (Shan et al., 2007; Wang et al., 2012). Briefly, 0.5–1.0 g fresh samples were ground in 80% methanol with butylated hydroxytoluene (1 mmol L^−1^) under an ice bath condition and transferred into a 10 ml centrifuge tube for 4 h at 4°C. After centrifugation, the supernatants were passed through a C18 Sep-Pak cartridge, washed with methanol or diethyl ether, and then dried in nitrogen. The residues were dissolved in phosphate-buffered saline solution (PBS) and transferred into 96-well microtitration plates and then coated with synthetic GA1-ovalbumin for 30 min at 37°C. After washing the plate with PBS four times, the horseradish peroxidase-labeled goat anti-rabbit immunoglobulins were added and incubated for 30 min at 37°C. After washing the plate with PBS, ortho phenylenediamine and H_2_O_2_ were added for coloration in the dark for 15 min at 37°C, and the reaction was stopped by adding 2 mol L^−1^ H_2_SO_4_. The absorbance was measured using the ELISA analyzer (Thermo Multiskan MK3) at 490 nm. Three biological replicates of each assay were performed.

## Results

### Isolation and sequence analysis of *VyMYB24*

**Figure 1:**
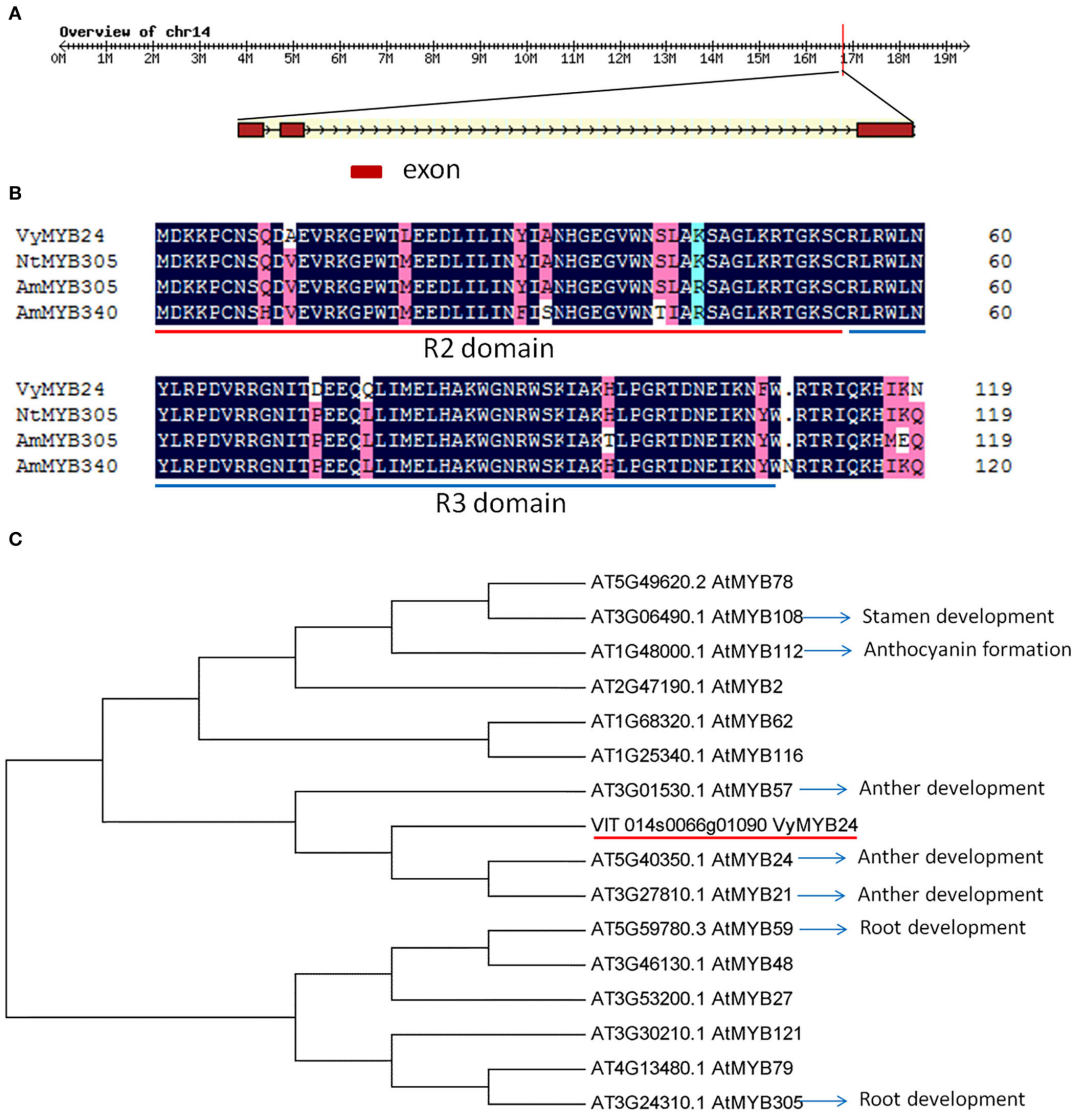
Features of VyMYB24 protein. **(A)** The genomic sequence of *VyMYB24*. **(B)** Alignment of VyMYB24 protein with other R2R3-MYB proteins from *N. tabacum* and *A. majus*. R2 and R3 are the two repeats of the MYB DNA-binding domain. GeneBank accession numbers are as follows: NtMYB305 (XP_016506729), AmMYB305 (P81391), and AmMYB340 (P81396). **(C)** Phylogenetic analysis of VyMYB24 protein with other 15 R2R3-MYB proteins from *Arabidopsis*. The phylogenetic tree was generated using the neighbor-joining method by MEGA5.0. Part of the R2R3-MYB genes was involved in plant development.

Using the PCR method, the full length of *VyMYB24* cDNA was cloned, and its open reading frame was 570 bp encoding 190 amino acids and 21.569 kD of the predicted protein molecular weight. Based on the genome of *Vitis vinifera* (https://www.genoscope.cns.fr/externe/GenomeBrowser/Vitis), the *VyMYB24* gene was located at 16764247–16767889 on Chromosome14, and the gDNA of 3643 bp contained three exons (136, 130, and 304 bp, respectively) and two introns (93 and 2980 bp) (Figure 1A). The protein sequence analysis showed that *VyMYB24* contained a conservative R2R3 domain, which had a high similarity of 78.65, 70.24, and 68.84% with NtMYB305, AmMYB305, and AmMYB340, respectively, from *Nicotiana tabacum* and *Antirrhinum majus* (Figure 1B). The phylogenetic analysis of VyMYB24 and 15 MYB proteins from *Arabidopsis* showed that VyMYB24 belonged to the same cluster as AtMYB24, AtMYB21, and AtMYB57, which were involved in the regulation of anther development (Cheng et al., 2009) (Figure 1C).

### *VyMYB24* gene was localized in the nucleus

**Figure 2:**
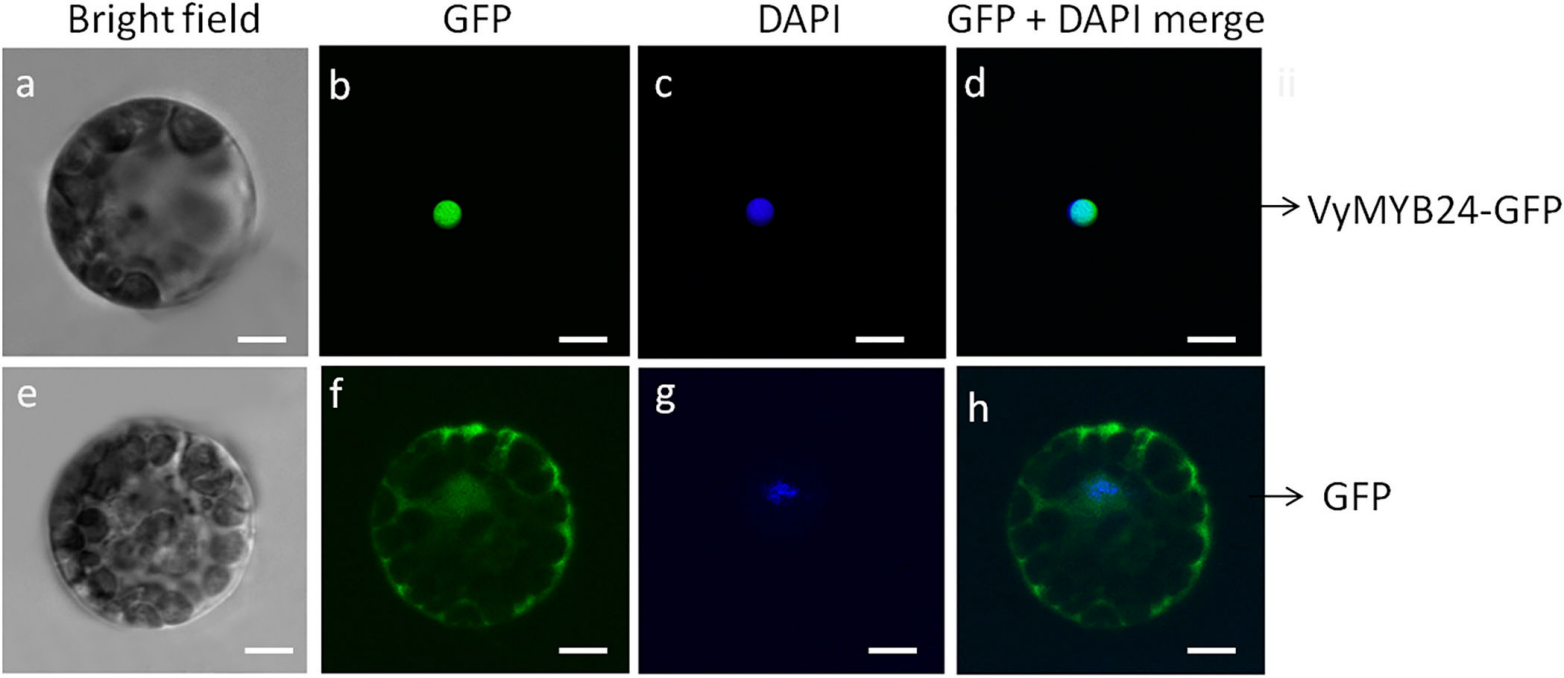
Subcellular localization of the VyMYB24 protein in *Arabidopsis* leaf protoplasts. The fluorescent signal was detected by confocal laser-scanning microscopy. **(a,e)** Bright field; **(b,f)** GFP; **(c,g)** DAPI; **(d,h)** GFP + DAPI merge. Bars correspond to 10 μm.

To confirm whether *VyMYB24* was localized in the cell nucleus, the plasmid pBI221-*VvMYB24-*GFP was transformed into *Arabidopsis* mesophyll protoplasts. The results showed that the VvMYB6-GFP fusion protein was located solely in the nucleus, whereas the control GFP protein was distributed throughout the cell, including the nucleus, the cytoplasm, and the cell wall (Figure 2), suggesting *VyMYB24* as a nuclear protein.

### *VyMYB24* caused dwarf phenotype in the transgenic tobacco plants

**Figure 3:**
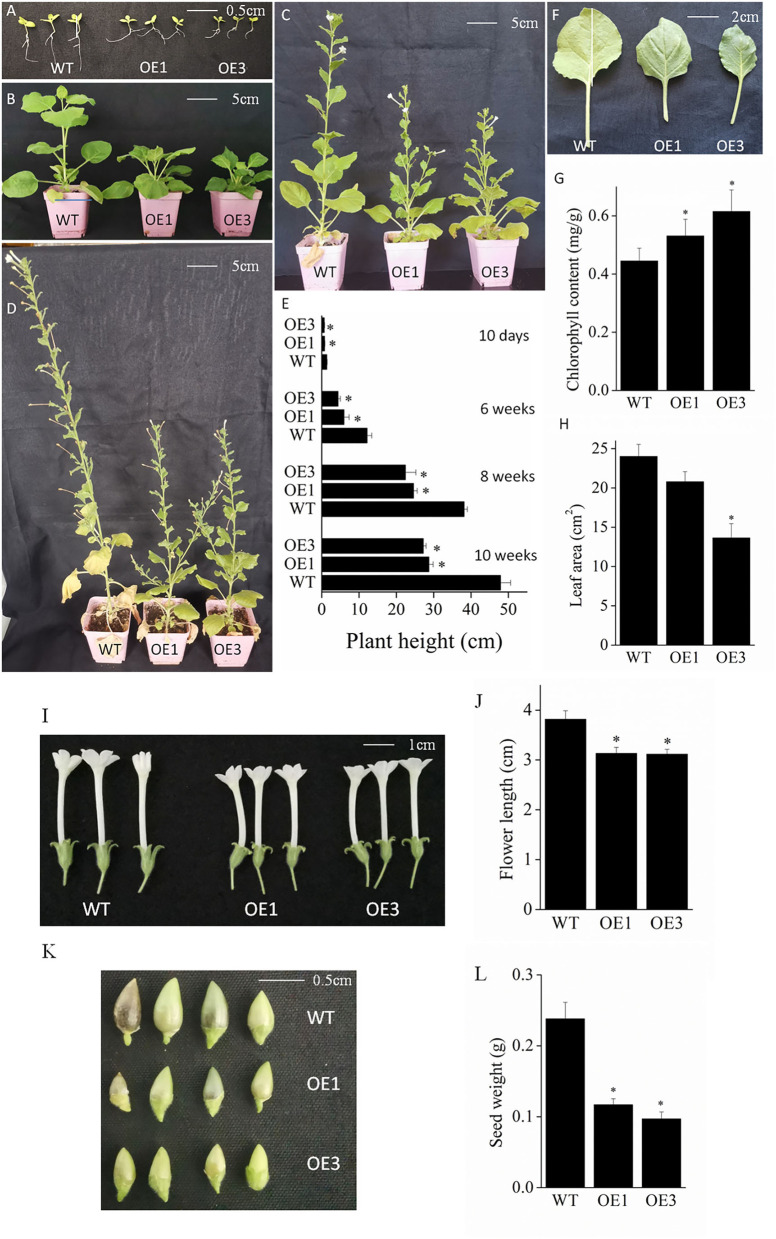
Phenotypic analysis of *VyMYB24* transgenic tobacco plants. The phenotypes of 10-day seedlings **(A)**, six-week plants **(B)**, eight-week plants **(C)**, and ten-week plants **(D)** were observed, and the plant height was measured **(E)**. Leaves from six-week plants **(F)** were chosen to measure the leaf area **(G)** and the chlorophyll content **(H)**. The flowers **(I)** and seeds **(K)** were photographed and the flower length **(J)** and seed weight **(L)** were evaluated. Bars represent mean ± SD. A significant difference from the WT was confirmed by a one-way ANOVA test, ^*^p < 0.05.

To further explore the potential function of the *VyMYB24* gene, transgenic tobacco plants were obtained. Compared with the wild-type plants, the transgenic *VyMYB24* tobacco lines (OE1 and OE3) all exhibited the dwarf phenotype at 2, 6, 8, and 10 weeks (Figures 3A–D). The height of transgenic plant lines OE1 and OE3 were 54.07 and 48.15% of wild-type tobacco plants at two weeks; it comparatively reduced to 49.01 and 35.83% at six weeks, increased to 64.44 and 58.73% at eight weeks, and reduced again to 59.91 and 56.78% of the wild-type plants at 10 weeks (Figure 3E). In addition, we also found that leaf areas were smaller in transgenic tobacco plants (Figures 3F,H); the leaf areas of the transgenic plant lines OE1 and OE3 were 86.56 and 56.76% of the wild-type plants at six weeks. The transgenic tobacco plants also accumulated more chlorophyll (Figures 3F,G), reaching 119.10% (OE1) and 138.22% (OE3) of the wild type. Meanwhile, shorter flowers and smaller seeds were observed in the transgenic *VyMYB24* lines (Figures 3I–L); the flower length of OE1 and OE3 were 82.15 and 81.50% of the wild-type plants and their seed weight were only 49.16 and 40.74% of wild type. In summary, the *VyMYB24* gene significantly modulated plant growth and development.

### *VyMYB24* gene regulated GAs biosynthesis in transgenic plants

**Figure 4:**
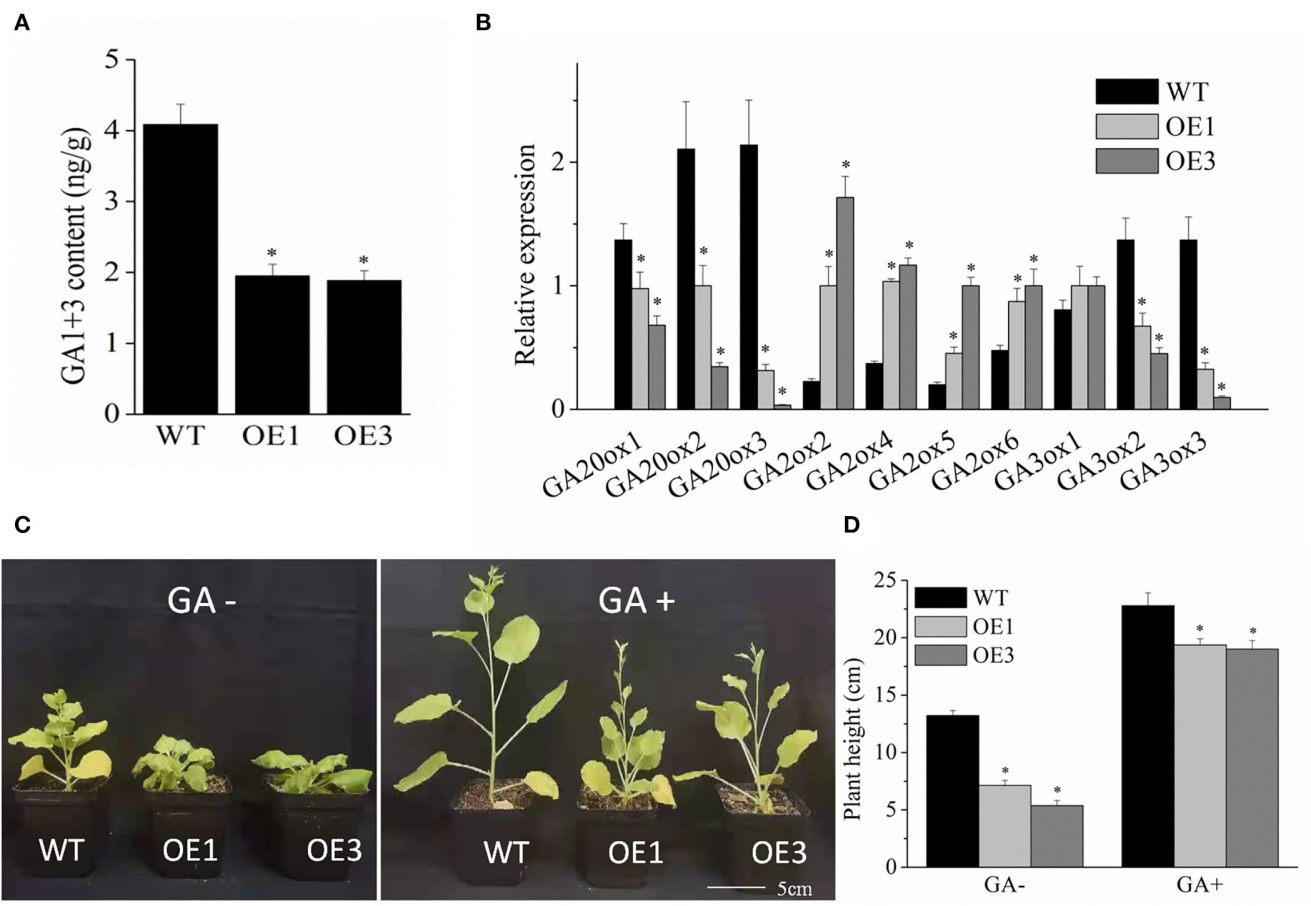
The *VyMYB24* gene regulated GA biosynthesis in the transgenic lines. **(A)** The content of GA1+3 was measured by the ELISA method. **(B)** Expression profiles of the GA biosynthesis-related genes. **(C)** Phenotypic characteristics of the transgenic lines and wild-type plants were sprayed with 10^−5^ M GA after three weeks, and the plant height was measured **(D)**. Bars represent mean ± SD. A significant difference from the WT was confirmed by a one-way ANOVA test, ^*^p < 0.05.

As an endogenous hormone, GA plays a key role in regulating plant height development. To examine the reason for the dwarf phenotype of a transgenic plant, the GA content was measured using the ELISA method. As shown in Figure 4A, GA1+3 content was significantly lower in the transgenic *VyMYB24* lines compared with the wild type. The GA1+3 content of transgenic plant lines OE1 and OE3 were 47.72 and 46.17% of that of wild type, respectively. Meanwhile, the GA metabolism-related genes were checked using the qRT-PCR method. As shown in Figure 4B, the genes related to GA synthesis (*GA20ox1, GA20ox2, GA20ox3, GA3ox2*, and *GA3ox3*) were down-regulated significantly in the transgenic lines except for *GA3ox1*, while the genes related to GA degradation genes (*GA2ox2, GA2ox4, GA2ox5*, and *GA2ox6*) were positively regulated significantly (Figure 4B). To further verify the above results, the phenotype recovery test was conducted. After exogenous spraying of GA for three weeks, the transgenic and wild-type tobacco plants all grew significantly taller (Figure 4C). However, the height difference between transgenic and wild-type plants were 15.1–16.7% under GA spraying conditions, which was markedly lesser than without GA spraying (46.9–59.4%) (Figure 4D), indicating that exogenous GA could recover the dwarfing phenotype of transgenic plants.

### Expression profiles of *VyMYB24* gene under stresses

**Figure 5:**
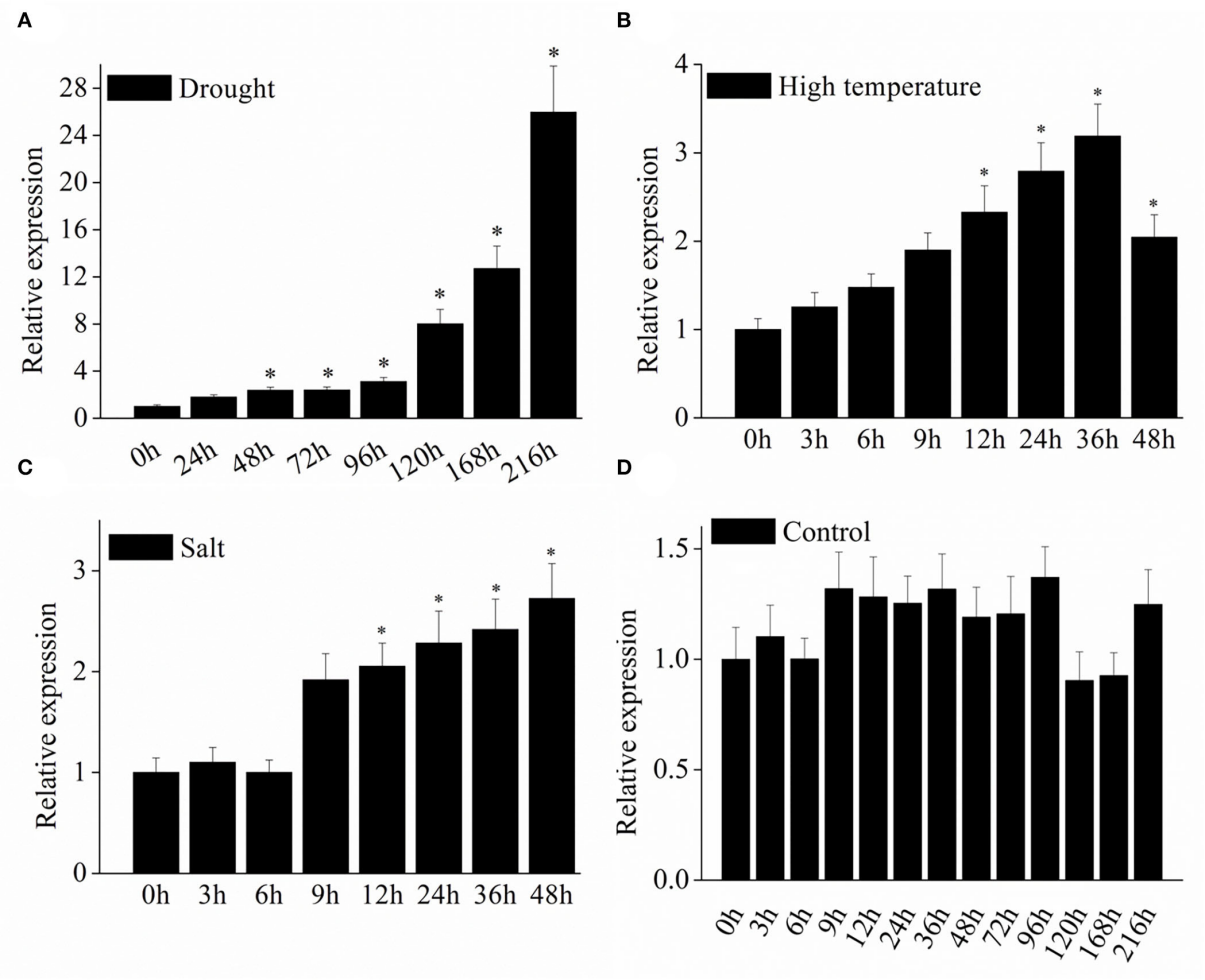
The expression profiles of the *VyMYB24* gene in *V. yanshanesis* accession Yanshan-1 leaves under abiotic stresses. The two-year-old Yanshan-1 seedlings were treated with drought **(A)**, high temperature of 40°C **(B)**, and high salt of 0.1 M NaCl **(C)** with normal as CK **(D)**. The data represents the means ± SD of three replicates and the statistical analyses were performed with a one-way ANOVA test, ^*^p < 0.05.

To determine the response of the *VyMYB24* gene to abiotic stresses, the gene expression was evaluated in grapevine leaves under drought, high temperature, and salt stresses using the qRT-PCR method. We found that the *VyMYB24* gene expression was induced by three abiotic stresses (Figure 2). The transcript abundance of *VyMYB24* reached its peak at 36 h, which was 3.18 times higher than at 0 h under high temperature, and 2.72 times higher at 48 h than at 0 h under salt stress. It was noted that the *VyMYB24* gene responded remarkably to drought stress (Figure 5) with 25.96 higher folds at 9 d than at 0 d, suggesting that the *VyMYB24* gene was greatly involved in the drought stress.

### *VyMYB24* gene enhanced the drought tolerance

**Figure 6:**
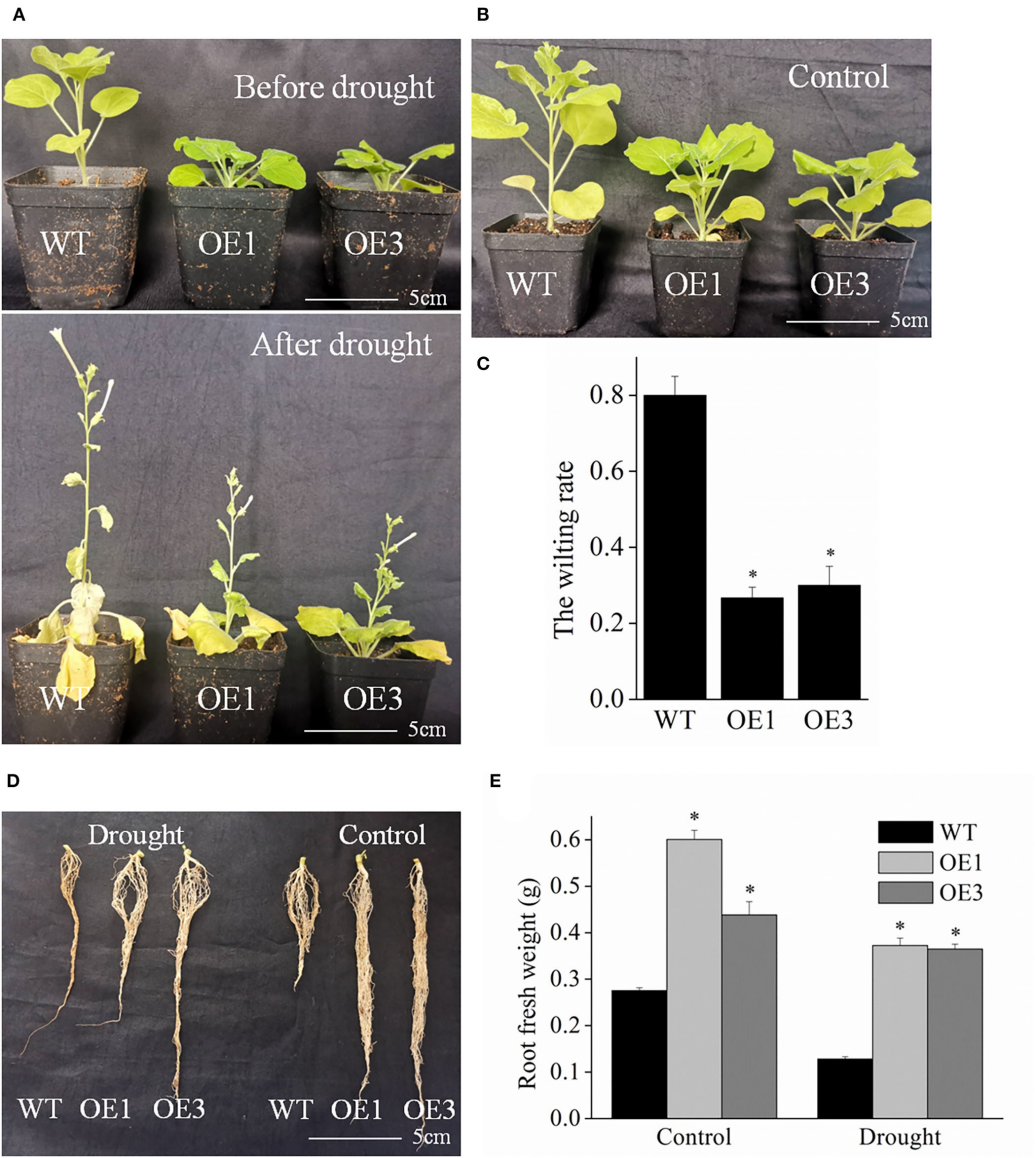
The *VyMYB24* gene improved the drought resistance in the tobacco transgenic lines. **(A)** Four-week-old transgenic and wild-type plants were treated with drought stress for 12 days with the normal as CK **(B)**. **(C)** The wilting rate of plants was measured, and the wilting rate was calculated based on the numbers of the plants with more than 50% leaves with wilting phenotype to the total plants. **(D)** The root phenotypes were observed before or after drought treatment, and the root fresh weight of plants was measured **(E)**. Bars represent mean ± SD. A significant difference from the WT was confirmed by a one-way ANOVA test, ^*^p < 0.05.

As seen in Figures 6A,B, at 12 d after drought stress, most of the wild-type tobacco plants displayed a serious wilting phenotype, while fewer transgenic plants exhibited wilting phenotype. The wilting rate of transgenic plants was 26.7–30%, which was significantly lower than that of wild-type plants (80%) (Figure 6C). Moreover, the transgenic plants had stronger roots (Figure 6D). Under normal watering, the root fresh weight of transgenic plant lines OE1 and OE3 was 0.60 and 0.44 g, but only 0.28 g for the wild-type plants. Drought stress hinders root growth in plants. However, our results demonstrated that the roots were stronger in the transgenic *VyMYB24* plants than in wild-type ones, and the root fresh weight of transgenic plant lines OE1 and OE3 were more by 2.85 and 2.77- folds in wild-type plants (Figure 6E). Collectively, the *VyMYB24* gene improved the drought tolerance in transgenic lines due to a lower wilting rate and stronger roots.

### ROS accumulation by histochemical staining

**Figure 7:**
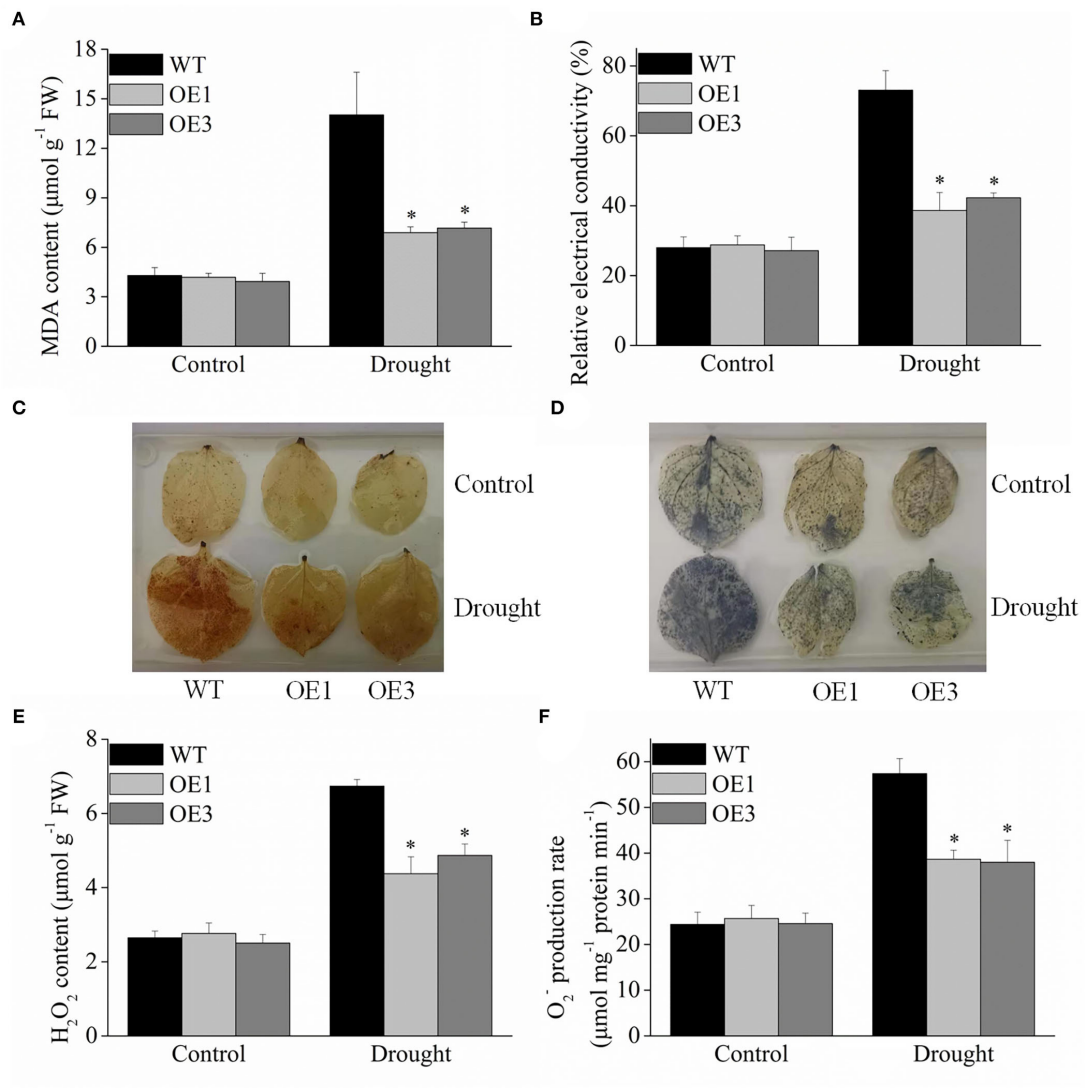
Oxidation conditions in *VyMYB24* transgenic and wild-type plants exposed to drought stress. Four-week-old tobacco plants were treated with drought stress for 10 days, and the MDA content **(A)** and relative electrical conductivity **(B)** were checked. **(C)**, **(D)** Detection of H_2_O_2_ and ${\text{O}}_{2}^{-}$ by DAB staining and NBT staining in transgenic and wild-type plants. H_2_O_2_ content **(E)** and ${\text{O}}_{2}^{-}$ production rate **(F)** were evaluated. Bars represent mean ± SD. A significant difference from the WT was confirmed by a one-way ANOVA test, ^*^p < 0.05.

MDA is an important physiological index to evaluate membrane lipid peroxidation. Under drought stress, the *VyMYB24* transgenic plant lines accumulated less MDA, about 54.07 and 48.15% lesser than wild-type plants (Figure 7A), indicating that the integrity of cell membrane was less destroyed in transgenic plants. Furthermore, the relative electrical conductivity was 0.38–0.42 in the transgenic plants, significantly lower than that of the wild-type plants, which was 0.73 under drought stress (Figure 7B).

DAB and NBT staining are commonly used to locate the H_2_O_2_ and ${\text{O}}_{2}^{-}$ in plant tissues. Under normal conditions, the tobacco leaves of the control appeared lighter in color with no significant difference between the transgenic and wild plants. Under drought treatment for 10 d, more yellowish-brown plaques were distributed on wild-type tobacco leaves compared to the transgenic plants using DAB staining (Figure 7C). When stained with NBT, the wild-type leaves appeared darker blue, while slight blue color was observed in the transgenic tobacco leaves (Figure 7D). Meanwhile, the H_2_O_2_ and ${\text{O}}_{2}^{-}$ contents were also evaluated. Under normal conditions, H_2_O_2_ and ${\text{O}}_{2}^{-}$ no significant differences were observed between the transgenic and wild-type plants. Under drought stress, the wild-type tobacco plants significantly accumulated more H_2_O_2_ and ${\text{O}}_{2}^{-}$, and the H_2_O_2_ content in the transgenic plant lines OE1 and OE3 was 64.93 and 72.57% of wild type (Figure 7E), and ${\text{O}}_{2}^{-}$ was 67.38 and 66.20% of the wild-type ones (Figure 7F).

### Analysis of antioxidant enzyme activity

**Figure 8:**
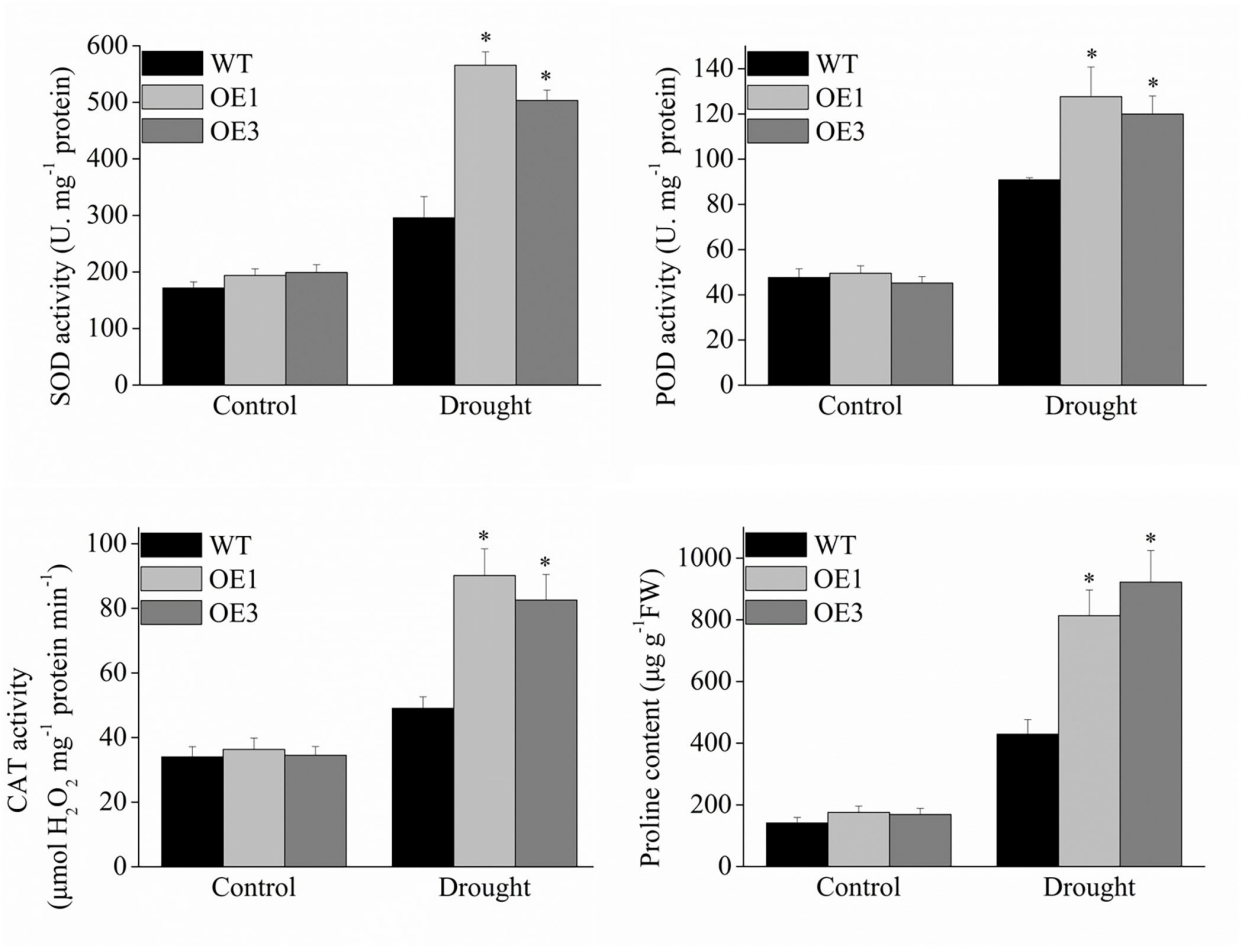
The total activities of the antioxidant enzymes and proline content in transgenic and wild-type plants under drought stress. Four-week-old tobacco plants were treated with drought stress for 10 days, and the activities of SOD, POD, CAT, and proline content were measured. Bars represent mean ± SD. A significant difference from the WT was confirmed by a one-way ANOVA test, ^*^p < 0.05.

Reactive oxygen-scavenging enzymes play an important role in plant response to environmental stresses. Under normal conditions, the enzyme activity of SOD, POD, and CAT were not significantly different between the transgenic and wild-type plants, However, the transgenic *VyMYB24* tobacco plants had a significantly higher enzyme activity compared to the wild-type ones under drought stress (Figure 8). The SOD activity in OE1 and OE3 was 191 and 170% of wild type ones, the POD activity was 140 and 132% of wild type, and the CAT activity was 184 and 168% of wild type. Meanwhile, proline was significantly accumulated in the *VyMYB24* transgenic plants under drought stress (Figure 8), especially in OE3 – two-folds higher than the wild-type ones.

### Expression profiles of the stress-related genes

**Figure 9:**
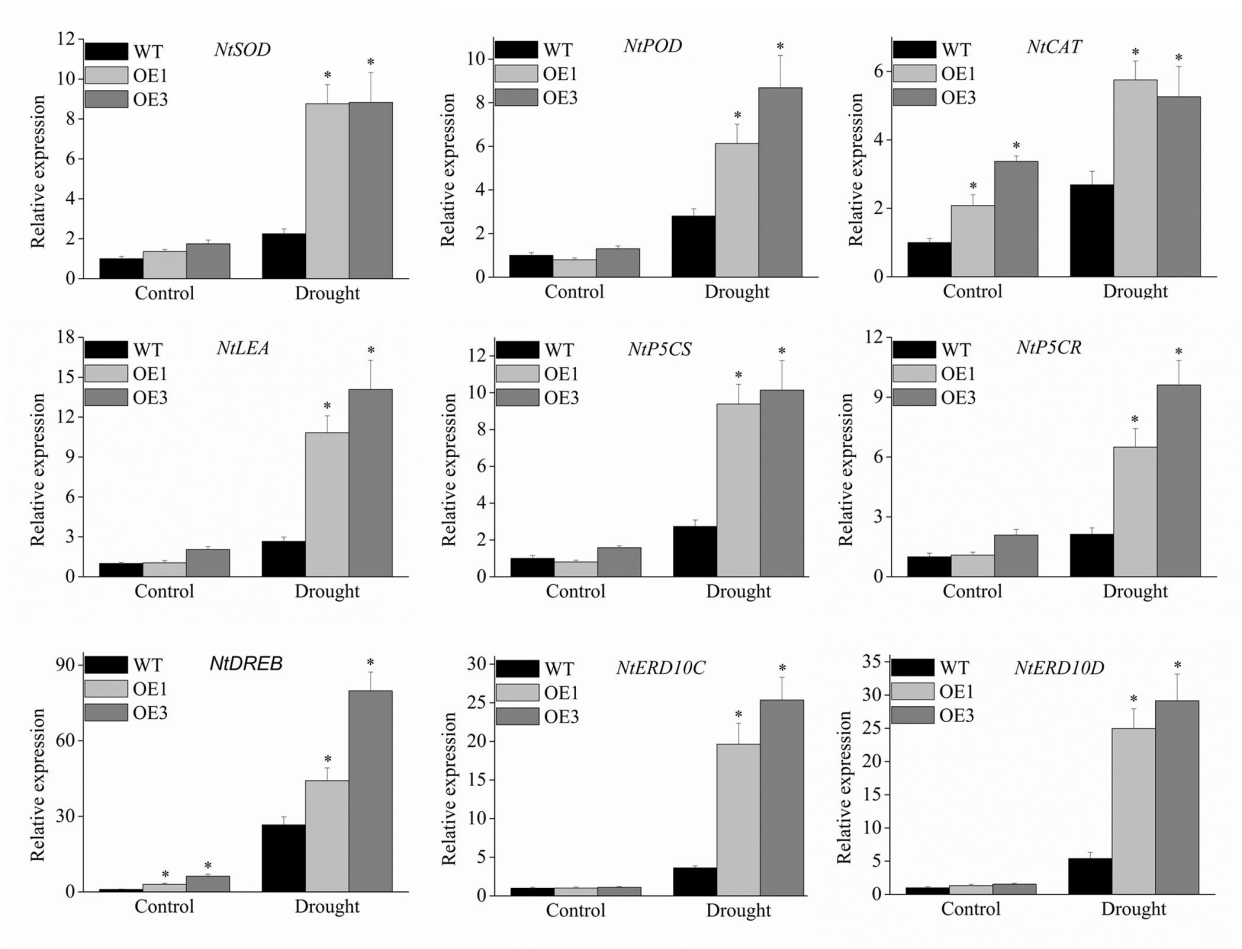
Expression profiles of the stress-responsive genes in transgenic plants under drought stress. The expression data were normalized against the expression of *NtActin*. Bars represent mean ± SD. A significant difference from the WT was confirmed by a one-way ANOVA test, ^*^p < 0.05.

To further explore the molecular mechanism underlying the enhanced drought resistance in transgenic lines, nine stress-related genes including *NtSOD, NtPOD, NtCAT, NtLEA5, NtP5CS NtP5CR, NtDREB, NtERD10C*, and *NtERD10D* were investigated using the qRT-PCR method. The results showed that all nine stress-related genes were all up-regulated by drought stress in transgenic lines compared to the wild type (Figure 9), especially, *NtERD10C, NtERD10D*, and *NtLEA5*.

## Discussion

Grapevine (*Vitis vinifera* L.), as a commercial horticultural crop, is cultivated worldwide. However, drought, as the major environmental stressor, seriously impacts the growth and development of the plant but more particularly affects berry and wine quality and grapevine yield (Costa et al., 2007; Chaves et al., 2010). Several reports demonstrate that MYB transcription factors regulate diverse plant developmental processes, affecting biotic and abiotic stresses (Liu et al., 2011; Lv et al., 2017; Zeng et al., 2019; Zhou et al., 2019; Lu et al., 2021). However, very little is known about how MYB transcription factors mediate the response to drought stress in grapevines. In the present study, the *VyMYB24* gene was obtained based on the transcriptome analysis of *V. yeshanensis* accession Yanshan-1 under drought stress. Sequence analysis showed that the VyMYB24 protein contained a conservative R2R3 domain and is localized in the nucleus of the *Arabidopsis* leaf protoplasts, which was in accordance with the typical characteristics of transcription factors (Stracke et al., 2001).

### *VyMYB24* Inhibited the Development of Transgenic Plants

Members of MYB from other species were involved in plant development, including organ morphogenesis, flower development, and plant growth (Cheng et al., 2009; Seo et al., 2009; Lau et al., 2015). Homology analysis of protein sequences showed that VyMYB24 has a high similarity to NtMYB305 from *N. tabacum* and AmMYB330 from *Antirrhinum* (Figure 1B), which could inhibit plant development in transgenic plants that displayed dwarf phenotype (Tamagnone et al., 1998; Liu, 2010). Our results further demonstrated that overexpressing *VyMYB24* caused plant dwarfing, resulting in growth parameters smaller than wild plants, including plant height, leaf area, flower length, and seed weight (Figure 3). A similar phenotype was also found in *Arabidopsis*, overexpressing the *MYB96* plants also exhibited dwarf growth, altered leaf morphology, and reduced lateral roots (Seo et al., 2009). In addition, the phylogenetic analysis showed that *VyMYB24* fell into the same cluster as *AtMYB24, AtMYB21*, and *AtMYB57* from *Arabidopsis*, which were involved in anther development (Cheng et al., 2009), but *VyMYB24* did not affect the development of anthers in transgenic plants, and no male sterile phenotype was found (Figures 3I,K). Therefore, it was inferred that *VyMYB24* was involved in just regulating plant height.

### *VyMYB24* enhanced the tolerance to drought

The expression analysis under different stresses showed that *VyMYB24* was induced significantly by drought stress compared to high temperature and salt stresses (Figure 5). This prompted us to explore the possible role of the *VyMYB24* gene under drought stress. Treated with drought treatment, transgenic plants displayed a lower wilting rate and lower relative electrical conductivity compared to the wild-type plants (Figure 6), suggesting that *VyMYB24* could improve the plant's tolerance to drought. Moreover, *VyMYB24* transgenic plants had more strong roots compared to wild-type plants. This result was similar to *MdSIMYB1* from *Malus Domestica*, where the heterologous expression of *MdSIMYB1* in tobacco enhanced the tolerance to drought and exhibited robust root growth (Wang et al., 2014). Similarly, apple gene *MdMYB88* transgenic plants had more roots and higher hydraulic conductivity under drought stress (Geng et al., 2018). The plant root is a critical organ that absorbs water and mineral nutrients from the soil. A developed root can absorb more water from the soil which contributes to drought resistance (Gullo et al., 1998; Hoecker et al., 2006). The above results suggested that strong roots might play a role in the drought resistance of *VyMYB24* transgenic plants.

Reactive oxygen can regulate plant development as a signal molecule, and the production and the elimination of reactive oxygen keep a balance under normal conditions. When exposed to stress, the plant will accumulate excess reactive oxygen, which causes membrane lipid peroxidation, resulting in membrane system damage and, even worse, the death of the plant (Gechev et al., 2006). Plants exhibit an active oxygen scavenging system. Through a series of enzymatic reactions, the SOD enzyme can convert ${\text{O}}_{2}^{-}$ into molecular oxygen, and POD enzyme and CAT enzyme can convert H_2_O_2_ into H_2_O and O_2_. Previous studies showed that many MYB genes such as from *Arabidopsis, OsMYB6* from rice (Tang et al., 2019), *GaMYB85* from cotton (Butt et al., 2017), *TaMYB33* from wheat (Qin et al., 2012), and *MdSIMYB1* from apple (Wang et al., 2014) could regulate the production of reactive oxygen by the antioxidant enzyme system. In the present study, *VyMYB24* transgenic plants produced less reactive oxygen compared with the wild-type plants under drought stress. Meanwhile, *VyMYB24* transgenic had increased SOD, POD, and CAT activity than wild-type plants, and *NtSOD, NtPOD*, and *NtCAT* genes were significantly upregulated. The above result suggested that *VyMYB24* might reduce the production of reactive oxygen by upregulation of antioxidant enzyme activity.

Under stress treatment, stress-related genes, such as *P5CR, P5CS, DREB, ERD*, and *LEA*, were significantly induced, which promoted the synthesis of protective enzymes or active downstream genes. In this study, *NtP5CR* and *NtP5CS* genes were upregulated significantly compared to wild-type plants, and more specifically, proline was accumulated in *VyMYB24* transgenic when exposed to drought. Proline plays an important role in osmotic regulation, which can hold water and prevent dehydration under drought stress (Hong et al., 2000). As key regulatory enzymes, P5CR and P5CS are involved in the biosynthesis of proline (Delauney and Verma, 1993). Many drought tolerance MYB genes also promote the accumulation of proline under drought stress, such as *GmMYBJ1* from soybean (Su et al., 2014), *FtMYB9* from Tartary buckwheat (Gao et al., 2017), *MdMYB10* from apple (Gao et al., 2011), and *SoMYB18* from sugarcane (Shingote et al., 2015). The DREB transcription factors belong to the AP2/EREBP family, which can combine with dehydration-responsive elements to activate downstream stress-related genes (Agarwal et al., 2006b). In *Arabidopsis*, overexpressing *DREB1A* in tobacco improved drought and cold stress tolerance and significantly activated the stress-related genes *NtERD10C* and *NtEED10D*, which are late embryogenesis abundant proteins (Kasuga et al., 2004). In this study, *NtDREB, NtERD10C, NtERD10D*, and *NtLEA5* genes were upregulated significantly in *VyMYB24* when exposed to drought stress. These results indicated that stress-related genes participated in the drought tolerance of *VyMYB24*.

### The role of GA in development and drought resistance

As an important hormone, GA plays a key role in regulating plant development and the reduction of GA levels and has been shown to contribute to plant growth restriction (Yamaguchi, 2008). In this study, the GA1+3 content was reduced significantly in transgenic plants (Figure 4A); meanwhile, exogenous GA spraying could recover the dwarf phenotype of *VyMYB24* transgenic plants (Figures 4C,D). Similar to the DREB transcription factor transgenic *GhDREB1* from cotton, GhDREB1 transgenic plants displayed a GA-deficient phenotype and could be rescued by exogenous GA3 application (Huang et al., 2009). During GA metabolism, regulation of the key genes of the GA biosynthetic or degradable pathway, such as *GA20ox, GA3ox*, and *GA2ox*, often results in different levels of bioactivity, followed by accelerated or retarded plant development. GA12 is converted to GA4 through oxidations on C-20 and C-3 by GA 20-oxidase (GA20ox) and GA3-oxidase (GA3ox), respectively, and GAs are metabolically deactivated by a class of GA 2-oxidase (GA2ox) (Yamaguchi, 2008). In *VyMYB24* transgenic plants, the GA synthesis-related genes were downregulated and the GA degradation-related genes were upregulated compared with wild-type (Figure 4B). The results were obtained in *Arabidopsis*, where the NAC transcription factor, *JUB1*, affects growth by negatively regulating genes encoding key enzymes of gibberellin *GA3ox1* (Shahnejat-Bushehri et al., 2016). Given the above results, *VyMYB24* might inhibit plant development by the regulation of GA metabolism.

Gibberellin plays a key role in response to environmental stress. When exposed to abiotic stress, such as drought, salt, or cold, there is a reduction of endogenous bioactive GAs (Achard et al., 2006; Magome et al., 2008). Inhibition of the biosynthesis of gibberellins (GAs) and the application of GAs to retardant-treated plants and GA-deficient mutants reverse their enhanced stress tolerance as well as their dwarf growth condition (Gilley and Fletcher, 1998; Vettakkorumakankav et al., 1999; Rademacher, 2000). In *Arabidopsis*, upregulation of the biosynthetic gene *GA20ox* or exogenous application of GA reduced the tolerance to drought, while downregulation of the biosynthetic gene *GA20ox* or *GA3ox* could enhance the tolerance to drought (Colebrook et al., 2014). Arabidopsis NAC transcription factor *JUB1*, which negatively regulated the biosynthesis of GA, increased the tolerance to drought, salt, and heat stress (Thirumalaikumar et al., 2018). So, we speculated that *VyMYB24* might enhance drought tolerance by negatively regulating GA biosynthetic pathway. Moreover, there is evidence that GA signaling may integrate multiple hormone signaling pathways in response to stress, such as the stress hormone jasmonic acid (Colebrook et al., 2014). Whether there are other hormones involved in drought tolerance of *VyMYB24* needs further study.

## Conclusion

In conclusion, grapevine *VyMYB24*, belonging to the R2R3-MYB gene, was first identified from *V. yanshanesis* with high drought tolerance. Specifically, the present study found that *VyMYB24* enhanced the tolerance to drought stress by stimulating strong roots and by regulating the antioxidant enzyme system, proline accumulation, and stress-related gene expression. Moreover, *VyMYB24* could inhibit plant development by negatively regulating the biosynthesis of GA. Therefore, the current study expanded the potential function of the grapevine MYB gene and provided new evidence to further explore the molecular mechanism of drought stress in the MYB gene family.

## Data availability statement

The original contributions presented in the study are included in the article/Supplementary material, further inquiries can be directed to the corresponding authors.

## Author contributions

ZZ, BL, and JS conceived and designed the experiment. ZZ and RQ conducted the experiment and data analysis. WX, GC, GY, XL, ZH, and GL contributed to the data analysis. ZZ and JS wrote the manuscript. WX, GC, GY, XL, ZH, GL, and BL drafted the discussion and revised the manuscript. All authors contributed to the article and approved the final version.

## Funding

This work was supported by the Major Science and Technology Innovation Major Project of Shandong Province (Grant No. 2019JZZY010727), the Shandong Province Natural Science Foundation of China (Grant No. ZR2020MC137), the Agricultural Improved Variety Project of Shandong Province (Grant No. 2020LZGC008), and the Key Science and Technology Project of Henan Province (Grant No. 222102110199).

## Conflict of interest

The authors declare that the research was conducted in the absence of any commercial or financial relationships that could be construed as a potential conflict of interest.

## Publisher's note

All claims expressed in this article are solely those of the authors and do not necessarily represent those of their affiliated organizations, or those of the publisher, the editors and the reviewers. Any product that may be evaluated in this article, or claim that may be made by its manufacturer, is not guaranteed or endorsed by the publisher.
