# Supplementary material for: An R2R3-MYB transcription factor VyMYB24, isolated from wild grape Vitis yanshanesis J. X. Chen., regulates the plant development and confers the tolerance to drought
Source: Front Plant Sci. 2022 Sep 8;13:966641. doi: 10.3389/fpls.2022.966641 (PMC9495713; doi:10.3389/fpls.2022.966641)
Supplement: Supplementary file 1 [file Table_1.docx]

Supplementary Material

# Supplementary Table 1 Primers used in this study for qPCR.

| Gene name | Forward Primer 5' → 3' | Reverse Primer 5' → 3' |
| --- | --- | --- |
| *VyMYB24* | aacgagattaagaatttctggagga | gagattggcagggtaggatggtgga |
| *VvActin* | cctcaaccccaaggccaacaga | accatcaccagaatccagcaca |
| *NtGA20ox1* | taggaggtttcctttctggagacc | gcgttggagatgagattagcgtta |
| *NtGA20ox2* | cagggcagtagtgaataaggagaag | aaaatggattaataagctgaggagg |
| *NtGA20ox3* | tgaggttccttcttcacagca | ctcccctaaaagctccattacc |
| *NtGA2ox2* | aatttgattggatttggtgagca | aatatttgaaggccggaagtgtt |
| *NtGA2ox4* | ccacctgatccaaattctttcttc | gtgccttacactcttaaacctccc |
| *NtGA2ox5* | atccagggtgtctatgatttatttt | caggttttgtttgtggtttcttttc |
| *NtGA2ox6* | ggaaggtttaaggacccaagaca | aacggtagggagaggaggagaga |
| *NtGA3ox1* | caccattgttggctcccctctt | ttttccatttccctttcgtatt |
| *NtGA3ox2* | gaagaatacgaaaaggagatgga | agaatttagttgtagggccgagc |
| *NtGA3ox3* | gatattgaattggcaggaaaaaa | cagtgacaccatcaggagaacga |
| *NtERD10C* | aacgtggaggctacagatcg | gttcctcttgggcatgagtt |
| *NtERD10D* | gaggacacggctgtaccagt | gcgccacttcctctgtctt |
| *NtP5CR* | agggagcttgcacttggac | gatgcttggccatacccttg |
| *NtP5CS* | gaacggaggttgctgatgga | tcccacttcggactgctaga |
| *NtPOD* | caaatgtaagaggaaactcagagg | agcaacaactccagctaattgatag |
| *NtSOD* | agctacatgacgccatttcc | ccctgtaaagcagcaccttc |
| *NtCAT* | aggtaccgctcattcacacc | aagcaagcttttgacccaga |
| *NtDREB3* | gccggaatacacaggagaag | ccaatttgggaacactgagg |
| *NtLEA5* | ttgaatctggggttttggtt | ggaagcattgacgagctagg |
| *NtActin* | aacagtttggttggagttctgg | catgaagattaaaggcggagtg |
